# Report on the conference on 'Men, Women, and Medicine: A New View of the Biology of Sex/Gender Differences and Aging' held in Berlin, 24–26th February 2006

**DOI:** 10.1186/1747-5341-1-11

**Published:** 2006-10-26

**Authors:** Antje Kampf

**Affiliations:** 1 Fachbereich Medizin, Institut für Geschichte, Theorie und Ethik der Medizin, Johannes Gutenberg-Universität Mainz, Am Pulverturm 13, 55131 Mainz, Germany

## Abstract

The first world wide symposium on the topic of gender-specific medicine provided the latest research on differences in sex and/or gender in medicine and medical care. The presentations ranged beyond the topic of reproduction to encompass the entire human organism. This report critically reviews three issues that emerged during the Conference: gender mainstreaming, the concept of sex/gender differences and the issue of men's health. It suggests that the interdisciplinary concept of gender-specific medicine has to be mirrored by the integration of social and cultural studies into medical research and practice.

## Background

This three-day conference, the first world-wide symposium on the topic of gender-specific medicine, attracted a wide range of distinguished researchers and clinicians. The focus of the Conference was to report on the latest research on sex and/or gender difference and its impact on physiological function, treatment procedure and patient outcomes. The presentations ranged beyond the topic of reproduction – the most obvious field of sex difference – to encompass the entire human organism, with the scheduled 92 papers and 98 poster presentations covering diverse medical sub-specialities. Individual papers can be accessed as abstracts in the journal *Gender & Medicine*, 3, Supplement A, 2006, or on the website [1]. This report focuses on three issues that emerged during the Conference: gender mainstreaming, concepts of sex and gender difference, and men's health.

## Gender mainstreaming and the implementation of practical guidelines

The concept of gender mainstreaming was originally established by OSAGI in 1997, and was later also adopted by the WHO. Gender mainstreaming calls for equitable attention to male and female examples in clinical research, treatment, education, and indeed the whole health care system. It aims to ensure recognition and understanding of gender in social policy processes, and thereby counteract gender inequality [2]. Gender mainstreaming has been widely discussed, particularly by experts in health policy, and the literature is considerable. For a critical overview of the concept and implementation of gender mainstreaming, see, for example [3]. The purpose of gender-specific medicine is to achieve these goals – in addition to real benefits to patient care. Yet gender-specific medicine is still used as a euphemism for women's health alone, and the distinct concepts of sex and gender are often not accepted as scientific categories in medical research and practice, despite their substantial relevance [4]. Nevertheless, the array of newly-emerging research, and the range of the papers presented at this conference, indicates a promising change of direction.

The last day of the conference saw a vigorous discussion on the dilemma of how to integrate gender mainstreaming into medical research and education, which is made particularly difficult as medical schools have taught gender blindness until now. Staff from institutions that already teach gender-specific medicine, including Columbia University (USA), Charité Universitätsmedizin (Berlin), Karolinska University Hospital (Stockholm) and Chiba Prefectoral Institute of Public Health (Japan), all offered their individual strategies for resolving this problem. Clinical research and hard data were needed to persuade colleagues to integrate gender-specific medicine into other medical sub-specialties. It was agreed that the transition from research to the application of such knowledge in patient care was difficult. While clinical researchers were preoccupied with identifying sex/gender differences in biomedicine, health care practitioners voiced their need for practical guidelines on how to institute gender-specific medicine in their offices. Some promising attempts to establish guidelines were presented, such as: at Monash University (Australia), where gender mainstreaming has been introduced to medical education; at the Office of Research on Women's Health, NIH (Bethesda, MD, USA) Institute, which has scheduled web-based training course on gender/sex medicine for 2006; and at the Charité, which also will commence teaching a European graduate course in gender-specific medicine this year. Finally, the establishment of an international society for gender-specific medicine was proposed.

## The conceptualisation of sex and gender difference

The way in which most papers dealt with the concepts of gender and sex is problematic. In contrast to the specificity of the medical data that was presented, particularity was often abandoned in relation to sex and gender, and the two concepts conflated. Or, as was the case for many papers, 'sex' (defined as biological fact) and 'gender' (defined as socially grounded) were presented (unwittingly or explicitly) as binary oppositions. Since at least the 1970s, mainly Western feminist and social theorists have criticised an oppositional conceptualisation of sex as opposed to gender, and nature as opposed to nurture, where the first term is privileged. Such a pattern of conceptualisation tended to define traits as solely biological (a 'universal given' [5]) and individual outlooks as predetermined, and this is understood to result in discriminatory social, cultural and medical practices. Since the 1990s, this biological and genetic essentialism is resurgent, this has been reflected most prominently by the Human Genome Project. (For a critical review of the project, see [6]). This tendency is exemplified by many papers at the Conference, and by the call for more research on sex/gender differences to be conducted on the genetic and molecular level. Although presenters emphasised that 'sex' had to be understood with reference to the environment, most papers were predicated on the notion that biological influences precede cultural and social influences. In this respect, the papers predominantly presented biological 'facts' without any attempt to integrate gender. Yet sex cannot be considered in isolation from the environment in which it necessarily occurs. As medical historian Thomas Laqueur has rightly noted, 'everything one wants to say about sex – however sex is understood – already has in it a claim about gender' – or is grounded in the environment or culture [7]. The problem with genetic and biological essentialism and medical care has been noted by Rieder and Lohff [4]. This was also acknowledged by the criticisms of medical students in attendance, who had expected less polarization of sex and gender concepts, and more discussion of the integration of social and cultural factors in gender-specific medicine.

Only a few papers presented an integrated concept of sex/gender; for example, a paper on the role of male and female health factors in stress, or a paper on the social and cultural factors imbedded in the clinical presentation of chronic pain [8,9]. Only one paper on 'Gender Bias in Medicine' specifically addressed the problems associated with the current approach to sex/gender distinctions [10]. The paper highlighted that a 'knowledge-mediated gender bias' was the outcome of an unquestioned use of the binary concepts of sameness/difference, inequity/equity, which resulted in the neglect of the individual patient in the clinical setting, in the case of gender-biased treatment for irritable bowel syndrome.

## The issue of men's health

Despite the conference title, only a few papers and poster presentations dealt explicitly with sex/gender difference in men. This may be a product of the Western feminist movement's campaign for the establishment of 'women's health' as a separate branch of medicine, to compensate for the limitations of the gender-neutral – male-orientated – norm in medicine. The legacy of this has been a perception of gender-specific medicine as 'women's medicine' with a political agenda. Of course, further research in the specific fields of women's health remains necessary, particularly as research for women is still restricted in scope due to concerns that clinical trials might affect women's fertility. However, there is a multitude of reasons that men's health should be a valid field of research, as was highlighted in 1999 by the first World Congress on Men's Health in Vienna [11]. The papers presented at this conference that were devoted to men's health drew attention to deficiencies in the health of men, and the need for further research [12,13]. Newer clinical research has also brought with it new methodological difficulties that restrict the applicability of medical findings for men. For example, it was noted that while more osteoporosis has been documented amongst women, more studies had been conducted on women in the first place; that while anorexia is a condition which is also found in men, this fact is often ignored, as it is considered a women's condition. The paucity of papers on topics related to men's health was mirrored by the paucity of male participants. The clear lack of male perspectives was regretted by the organisers and participants, and it was hoped that this problem could be remedied at the next conference by a stronger focus on men's topics.

## Conclusion

The Conference was not the first to examine the neglected question of sex and gender difference in medical research and treatment. Yet by presenting a wide range of the latest international medical research, the Conference drew attention to the capacity of this as yet little-understood field of research, in time, to reshape medical understanding. A further conference is scheduled in 2007, and the organisers intend a stronger integration of the social sciences as a separate discussion stream, and more medical studies on men. This is commendable, for as this Conference has demonstrated, it is clear that the interdisciplinary concept of gender-specific medicine calls for the integration of social and cultural studies into medical research and practice. While the American Institute of Medicine of the National Academies in 2001 stated that 'every cell has a sex' - the emphasis here was on the sexual genotype and chromosomal differences [14] - Dr John Bancroft (Senior Research Fellow at the Kinsey Institute for Research in Sex, Gender and Reproduction, Oxfordshire, U.K.) rightly reminded participants that bodies are not just cells, or given facts, but are equally influenced and shaped by culture and social factors [15]. Rather than using the concepts of gender mainstreaming and gender-specific medicine as empty vessels for biomedical research, the health care system should employ all the complementary biological, cultural and individual aspects of these concepts.

## Abbreviations

OSAGI Office of the Special Advisor on Gender Issues and Advancement of Women, United Nations

WHO World Health Organisation

NIH National Institute of Health

## Competing interests

The author(s) declare that they have no competing interests.
